# Characterization and colonization of endomycorrhizal *Rhizoctonia* fungi in the medicinal herb *Anoectochilus formosanus* (Orchidaceae)

**DOI:** 10.1007/s00572-014-0616-1

**Published:** 2015-01-11

**Authors:** Jr-Hau Jiang, Yung-I Lee, Marc A. Cubeta, Lung-Chung Chen

**Affiliations:** 1 Department of Plant Pathology, National Chung Hsing University, No. 250, Kuo-Kuang Road, Taichung, Taiwan; 2 Botany Department, National Museum of Natural Science, No. 1, Kuan-Chien Road, Taichung, Taiwan; 3 Department of Plant Pathology, Center for Integrated Fungal Research, North Carolina State University, Raleigh, NC 27695 USA

**Keywords:** Orchid mycorrhizae, *Rhizoctonia*, Anastomosis group, Symbiotic germination, Tissue-cultured seedling

## Abstract

The medicinal effects and techniques for cultivating *Anoectochilus formosanus* are well-documented, but little is known about the mycorrhizal fungi associated with *A. formosanus. Rhizoctonia* (*Thanatephorus*) anastomosis group 6 (AG-6) was the most common species isolated from fungal pelotons in native *A. formosanus* and represented 67 % of the sample. *Rhizoctonia* (*Ceratobasidium*) AG-G, P, and R were also isolated and represent the first occurrence in the Orchidaceae. Isolates of AG-6, AG-R, and AG-P in clade I increased seed germination 44–91 % and promoted protocorm growth from phases III to VI compared to asymbiotic treatments and isolates of AG-G in clade II and *Tulasnella* species in clade III. All isolates in clades I to III formed fungal pelotons in tissue-cultured seedlings of *A. formosanus*, which exhibited significantly greater growth than nonmycorrhizal seedlings. An analysis of the relative effect of treatment (${\widehat{p}}_i$) showed that the low level of colonization (${\widehat{p}}_i = 0.30\hbox{--} 0.47$) by isolates in clade I resulted in a significant increase in seedling growth compared to isolates in clades II (0.63–0.82) and III (0.63–0.75). There was also a negative correlation (*r* = −0.8801) with fresh plant weight and fungal colonization. Our results suggest that isolates in clade I may represent an important group associated with native populations of *A. formosanus* and can vary in their ability to establish a symbiotic association with *A. formosanus*. The results presented here are potentially useful for advancing research on the medicinal properties, production, and conservation of *A. formosanus* in diverse ecosystems.

**Electronic supplementary material:**

The online version of this article (doi:10.1007/s00572-014-0616-1) contains supplementary material, which is available to authorized users.

## Introduction

The genus *Anoectochilus* (Orchidaceae), which comprises more than 40 species, is widespread throughout tropical regions. Several species of this genus are used in Chinese folk medicines, such as *Anoectochilus formosanus* Hayata and *A* *noectochilus koshunensis* Hayata, which are terrestrial orchids and distributed only in Taiwan and Okinawa (Japan) (Gutiérrez 2010).

Because of its ornamental value and medicinal properties (Tseng et al. 2006; Wu et al. 2007), the number of native *A. formosanus* has been greatly decreased due to increased harvesting by intentional picking and uprooting from native habitats (Cheng and Chang 2009). *A. formosanus* collected from the wild are often sold for more than 300 USD per kilogram (Chang et al. 2007). Using plant tissue techniques (Ho et al. 1987), the propagation and cultivation of *A. formosanus* has been established in Taiwan for at least 20 years. However, pot culture and transplantation of the tissue-cultured plantlets in soil without colonized mycorrhizal fungi have been challenging due to poor plant growth and increased susceptibility to mites (Chang et al. 2007) and plant pathogenic fungi *Fusarium oxysporum* f. sp. *anoectochili* (Huang et al. 2014) and the oomycete species of *Pythium* (e.g., *P* *ythium aphanidermatum*, *Pythium myriotylum*, and *Pythium splendens*) (Chang 1999) during ex vitro growth. Therefore, we hypothesized that plant survival could be increased by inoculating seeds or tissue-cultured plantlets with mycorrhizal fungi. However, there is a paucity of knowledge regarding the mycorrhizal associations between native *A. formosanus* and its associated mycorrhizal fungi.

The beneficial association of symbiotic endomycorrhizal fungi with orchids is well-documented (Rasmussen 2002). In nature, the seeds of orchids are minute and contain few stored food reserves, and colonization by a mycorrhizal fungus provides nutrients that are important for seed germination and seedling establishment (Dearnaley 2007). In either green or in achlorophyllous species of orchids, the dependency on mycorrhizal fungi can continue into adulthood (Gebauer and Meyer 2003; Julou et al. 2005; Rasmussen and Rasmussen 2007). Recently, the applications of mycorrhizal association for horticultural and conservation purposes have gained considerable attention (Zettler et al. 2007; Swarts and Dixon 2009).

With few exceptions (Selosse et al. 2004), the majority of orchid mycorrhizal (OM) fungi belong to early diverging lineages in the phylum Basidiomycota (Moncalvo et al. 2006; Smith and Read 2008). Rasmussen (2002) suggested that photosynthetic orchids associate with a wide range of *Rhizoctonia* species. The form-genus *Rhizoctonia* D.C. includes *Thanatephorus* Donk, *Ceratobasidium* D.P. Rogers, *Tulasnella* Schröeter, and *Sebacina* Tul. (Sneh et al. 1991; González García et al. 2006; Smith and Read 2008). The specific association of photosynthetic orchids with *Rhizoctonia* fungi in the Tulasnellaceae and Ceratobasidiaceae (e.g., cantharelloid clade) has been previously reported (Otero et al. 2002; Ma et al. 2003; Suarez et al. 2006). Certain photosynthetic orchids, even when sampled over a wide range, have a single dominant species of *Rhizoctonia* fungus (McCormick et al. 2004; Shefferson et al. 2005, 2010; Irwin et al. 2007). However, fully mycoheterotrophic (MH) orchids, which are achlorophyllous and nutritionally dependent on their mycorrhizal fungi, can be colonized by several different ectomycorrhizal (ECM) fungi (e.g., Russulaceae and Thelephoraceae fungi) (Roy et al. 2009; Kennedy et al. 2011) as well as saprobic and parasitic fungi (Smith and Read 2008). By contrast, fully MH orchids revealed associations with more diverse fungal lineages (Dearnaley et al. 2012). Recently, certain reports have highlighted that *Rhizoctonia* fungi also have the ability to associate with orchids as OM fungi and with autotrophic plants as ECM fungi at the same time to form a tripartite symbiosis (Yagame et al. 2008, 2012; Bougoure et al. 2009).

The mycorrhizal specificity of orchids with *Rhizoctonia* fungi has been the subject of debate for many years (Leake 2005). Molecular and sequence-based analyses have provided an efficient and rapid means of characterizing and identifying strains of endomycorrhizal *Rhizoctonia* fungi that associate with orchids (Otero et al. 2002; Ma et al. 2003; Suarez et al. 2006; Taylor and McCormick 2008). Phylogenetic analysis of the fungal ribosomal DNA-internal transcribed spacer (rDNA-ITS) sequences has also suggested that both binucleate *Rhizoctonia* spp. (teleomorph = *Ceratobasidium*) and multinucleate *Rhizoctonia* spp. (teleomorph = *Thanatephorus*) can be divided into different anastomosis groups (AGs), which are determined by examining macroscopic somatic hyphal interactions between a representative tester strain and an unknown *Rhizoctonia* isolate (Carling et al. 1999; Sharon et al. 2008).

The primary objective of this study was to characterize and identify *Rhizoctonia* fungi sampled from fungal pelotons in *A. formosanus* collected from native populations in different geographic regions of Taiwan. Subsequent experiments were conducted to determine if these fungi promoted seed germination and seedling growth of *A. formosanus*. Furthermore, we investigated the degree of colonization caused by endomycorrhizal fungi under an in vitro condition. No information is currently available on the effects of the density of colonized endomycorrhizal fungi in *A. formosanus*. The results obtained from this study provide information on the characterization and colonization of endomycorrhizal fungi to *A. formosanus* and represent potentially useful data for medicinal orchid production and conservation in native habitats.

## Materials and methods

### Isolation of endomycorrhizal fungi

Samples of native *A. formosanus* collected from seven sites in northern and central Taiwan were investigated for mycorrhizal fungi. Locations of plants, sampling dates, and original sources of the plant samples are presented in Table 1.

**Table 1:** Representative isolates of *Rhizoctonia* fungi from fungal pelotons in *A. formosanus*, showing sampling dates, number of plant individuals and root samples, and locations of plants

Isolate	GenBank accession	Sampling dates	Plant individuals/total root samples	Locality^a^
ANOF 0	KJ495962	15 July 2010	3/6	YT
ANOF 2	KJ495964	14 November 2010	3/8	YN
ANOF 3	KJ495965	25 November 2010	3/8	ST
ANOF 4	KJ495966	22 February 2011	5/14	WH
ANOF D1	KJ495967	23 February 2011	5/16	WH
ANOF D2	KJ495968	12 March 2011	3/9	JH
ANOF 6	KJ495969	16 June 2011	4/14	PN
ANOF 7	KJ495970	10 July 2011	2/6	AC
ANOF G2	KJ495971	17 July 2011	2/5	AC

*YT* Yangmingshan in New Taipei City, *ST* Sanxia District in New Taipei City, *WH* Wufong Township in Hsinchu, *JH* Jianshi Township in Hsinchu, *YN* Yuchi Township in Nantou, *PN* Puli Township in Nantou, *AC* Alishan in Chiayi County

^a^Locations are indicated by codes

Each individual of *A formosanus* was divided into three separate parts: the roots, rhizome, and junction between root and rhizome. Sections of cortex tissue with fungal pelotons were excised, surface-disinfested in a 1 % solution of NaOCl for 1 min, rinsed in sterile water, and transferred to tap water agar (TWA) and potato dextrose agar (PDA) acidified with 0.2 % lactic acid to inhibit growth of bacteria. After incubation for 24–48 h at 24–26 °C, cortical cells were examined daily for the presence of fungal growth, and hyphal tips were excised and transferred to a fresh plate of PDA for morphological identification and to potato dextrose broth (PDB) to generate mycelium for subsequent DNA extraction and molecular identification. In addition, tissues with fungal pelotons were stored in 1.5-ml Eppendorf tubes at −80 °C for DNA extraction.

### Morphological characterization

Isolates with hyphal characteristics of *Rhizoctonia* fungi (e.g., branching near the septum of the cells in vegetative hyphae, constriction of hyphae and formation of a septum at a short distance from the point of origin, and absence of clamp connections) were cultured on PDA at 25 °C in the dark (Sneh et al. 1991). Hyphae from each isolate were placed in a solution consisting of one drop of 0.5 % (*w*/*v*) Safranin O and one drop of 3 % KOH (Bandoni 1979) and examined with a compound light microscope with an attached digital camera to determine the number of nuclei per hyphal cell (Carling et al. 1999).

### Seed harvest and symbiotic germination

Initially, six plants of *A. formosanus* Hayata from Wufong (Hsinchu) were cultured in a greenhouse at the National Chung Hsing University. Artificial cross-pollination was performed in six individual plants at the beginning of December 2011 and 2012. The pollinated inflorescences were covered with nylon mesh (48 μm) to avoid invasion of insects into the capsules. Capsules were harvested from 50 days after pollination. One capsule from each of three randomly selected plants was collected.

Seeds from the collected fruits were sown on the same day to avoid loss of viability. Capsules were surface-disinfested in 1 % NaOCl with 0.3 % of Tween 80 for 15 min, rinsed three times in sterile distilled water, and split in half with a sterile scalpel. Seeds were picked out of the capsules with sterilized forceps, and approximately 50 to 120 seeds were added to the surface of 0.25 % oatmeal medium (OMA) in a 6-cm-diameter Petri dish (Warcup 1981). A 1-cm^3^ block of mycelium obtained from an actively growing culture was placed in the center of the Petri dish on the surface of OMA. Each plate was inoculated with an isolate of *Rhizoctonia* fungi sampled from *A. formosanus* and also from other native orchids, including isolate Cno3-2 (accession no. JX514374 from *Zeuxine* sp.), Cno10-3 (accession no. JX514376 from *Cheirostylis hungyehensis*), CalS1-2 (accession no. JX514384 from *Calanthe sylvatica*), Eno3-3 (accession no. JX514377 from *Goodyera procera*), Sno6-1 (accession no. JX514393 from *Cymbidium ensifolium*), Sno3-3 (accession no. JX514385 from *Liparis nakaharai*), Sno5-12 (accession no. JX514386 from *Cleisostoma paniculatum*), and R02 (from *Calanthe arisanensis* kindly provided by Chang, Doris CN) that served as controls for the experiments. Modified Hyponex medium (Hy) (Chang and Chou 2001) and 1/2 strength Murashige-Skoog (MS) media (1962) were also used for asymbiotic seed germination and to assess seed vigor. Five replicates of each treatment were prepared for each isolate, and the experiment was repeated once. Plates were sealed with parafilm and incubated in darkness at 25 °C for 30 days to promote seed germination and initial protocorm development.

After 10 days of culture, seeds in the symbiotic and asymbiotic treatments were collected from the surface of agar plates, fixed overnight at 4 °C in 2 % paraformaldehyde plus 2.5 % glutaraldehyde in 0.2 M phosphate buffer at pH 7.0, rinsed three times in phosphate buffer, and placed in 1 % OsO_4_ for 2 h at 4 °C. Tissue was rinsed in phosphate buffer, dehydrated in a graded ethanol series, and embedded in LR white resin. For staining of prepared tissue sections, sections (1 μm) were stained with periodic acid-Schiff’s (PAS) reagent for bright-field microscopy. Carbohydrates (e.g., cellulose, starch, and glycogen) stain purplish red and nuclei may stain light pink (Ruzin 1999).

Germination (%) was determined 30 days after seed sowing. The process of symbiotic development from seed germination to protocorm development with differentiated shoots was divided into seven phases (Fig. 3) as follows: 0 = an undifferentiated embryo in the testa; I = embryo absorbed water and had slight swelling in the testa; II = embryo swelling was observed with the production of epidermal hairs that ruptured the testa; III = increased embryo swelling and rupturing of the testa that was <1,000 μm in length; IV = embryo was between 1,000 and 2,000 μm in length; V = embryo was between 2,000 and 3,000 μm in length; and VI = embryo length >3,000 μm. The emergence of the swollen embryo from the seed coat was used as the primary criterion to define seed germination (phase III).

### DNA extraction, rDNA-ITS sequencing, and phylogenetic analyses

For DNA extraction, tissues with fungal pelotons and mycelium of each isolate from liquid culture were frozen at −80 °C before use. DNA was extracted from the frozen samples using the DNeasy Plant mini kit (Qiagen, Hilden, Germany). Total DNA was recovered in 50 μL of distilled deionized water or TE buffer and stored at −20 °C. The fungal rDNA-ITS sequence (ITS1-5.8S-ITS2) was amplified using primer sets ITS1/ITS4 (White et al. 1990) and ITS1-OF/ITS4-OF (Taylor and McCormick 2008).

PCR reactions were performed using 50 pM of each primer, 500 ng of template DNA, 2.5 units of *Taq* polymerase, 200 mM each of dNTPs, and 1.5 mM of MgCl_2_. Reactions were performed in a GeneAmp^R^ PCR System 2700 (Applied Biosystems, Foster City, CA, USA) under the following thermoprofile: initial denaturation at 94 °C for 2 min, followed by 30 cycles of denaturation at 94 °C for 40 s, annealing at 55 °C for 60 s, extension at 72 °C for 60 s, and final extension at 72 °C for 5 min. PCR products were purified using the MinElute PCR Purification Kit (Qiagen). Clean samples were eluted with 50 μL sterile water and quantified for conducting sequencing reactions by using a NanoDrop^TM^ 1000 Spectrophotometer (ND-1000, Thermo Fisher Scientific, Waltham, MA). Cycle sequencing was conducted using BigDye version 3.1 chemistry, and sequencing was done on an ABI 3100 Genetic Analyzer (Applied Biosystems). Both strands of DNA were sequenced. The sequences obtained in this study are available from GenBank under the provided accession numbers (Table 1 and Supplemental data 1).

Sequences were subjected to a BLAST search (Altschul et al. 1979) using the National Center for Biotechnology Information database (http://www.ncbi.nih.gov/BLAST). Well-characterized and closely related taxa obtained from BLAST searches were also included in the phylogenetic analyses. Multiple sequence alignments of each ITS sequence were made with ClustalW (Thompson et al. 1994) packaged with BioEdit Sequence Alignment Editor (Ibis Biosciences, Carlsbad, CA, USA). Phylogenetic trees were generated using maximum parsimony (MP) analysis (MEGA 5) (Tamura et al. 2011). Genetic distances of MP search method were calculated using the Subtree-Pruning-Regrafting (SPR). One thousand bootstrap replicates were performed to estimate the node reliability of the phylogenetic trees. Bootstrap values >70 % were considered significant and represented well-supported groups (clades).

### Colonization of endomycorrhizal fungi in tissue-cultured plantlets

A seed-derived *A. formosanus* plant with a well-developed rhizome and shoot was selected (Shiau et al. 2002), and leaves and roots were removed from the plant. A single rhizome was cut into several lengths (1.5 cm), and each rhizome contained a node that had the potential to differentiate and produce a new seedling. Each small rhizome was placed on the surface of MS medium containing 0.5 ppm 1-naphthaleneacetic acid (NAA) and 3 ppm 6-benzylaminopurine (BA) at 24 °C in the dark for 30 days (Ho et al. 1987). Tissue-cultured seedlings with uniform size (~1.5–2 cm) produced from each node were chosen for colonization experiments, and the asymbiotic (control) and symbiotic treatments were arranged in a completely randomized design with eight replicates of each treatment. The fungus was inoculated onto the center of a slant tube containing 0.25 % OMA (Warcup 1981) and allowed to colonize the entire medium before each tissue-cultured seedling was transferred to the base of the slant tube. There were eight replicate slant tubes per treatment and the experiment was repeated twice. Slant tubes were incubated in a growth chamber (12 h day/night photoperiod and daytime illuminance of approximately 3,500 lx corresponded to ca. 47.25 μmol m^−2^ s^−1^ using cool white fluorescent lamps) at 25 °C for 4 months (Ho et al. 1987; Shiau et al. 2002).

After 4 months, tissue-cultured seedlings were removed from each slant tube, and agar was carefully removed from the surface of the rhizome and roots and weighed to determine fresh weight. Generally, each plantlet had four to eight nodes. For our experiments, we randomly selected four plantlets in each of the fungal treatments to examine each node for fungal pelotons. Each node of an *A. formosanus* rhizome containing a branching root (>3 mm) was scored based on the colonized percentage of the entire surface (fungal pelotons) using category values from 0 to 4 (Fig. 6), where 0 = no fungal pelotons were observed, 1 = cortex was slightly colonized and formed a few pelotons at the junction of the rhizome and branching root; 2 = more fungal pelotons formed at the upper and lower site in the branching root; 3 = the basal part of the branching root (1–3 mm) was full of fungal pelotons, which have spread to the rhizome; and 4 = fungal pelotons were full of branching root and rhizome. From each root or rhizome with fungal colonization, a small piece of tissue (0.5 cm in length) was excised and macerated in 1 ml of sterilized distilled water with a sterilized glass rod to isolate the intracellular fungal pelotons. Individual pelotons were collected and plated on TWA and PDA to isolate the fungus.

## Statistical analysis

The experimental data in mycorrhizal synthesis of tissue-cultured seedlings experiments were analyzed using SAS software version 9.4 (SAS Institute Inc., Cary, NC). The ordinal data of a 0-to-4 scale for fungal colonization were analyzed using the nonparametric methodology of Brunner et al. (2002) as described by Shah and Madden (2004). Rank assigned to each observation was determined by PROC RANK and then PROC MIXED to calculate test statistics and levels of significance. Estimated relative treatment effects were calculated using ${\widehat{p}}_i = 1/N$ (${\overline{R}}_i - 1/2$) in which values are between 0 and 1, where *i* is the treatment, *N* is the number of observations, and $\overline{R}$ is the mean rank. Ninety-five percent confidence intervals (CI) were generated using LD_CI macro (Brunner et al. 2002). A smaller value of ${\widehat{p}}_i$ for a treatment indicates lower value for the degree of fungal colonization. Values of ${\widehat{p}}_i$ between two treatments are significantly different if the CI for each ${\widehat{p}}_i$ does not overlap.

Data on plant weight were analyzed by analysis of variance (ANOVA). Mean separation was performed by protected post hoc Tukey’s test (*P* = 0.05) after a significant *F* test in SAS ver. 9.4 (SAS Institute, Cary, NC) was determined.

## Results

### Endomycorrhizal fungi from *A. formosanus*

**Fig. 1:**
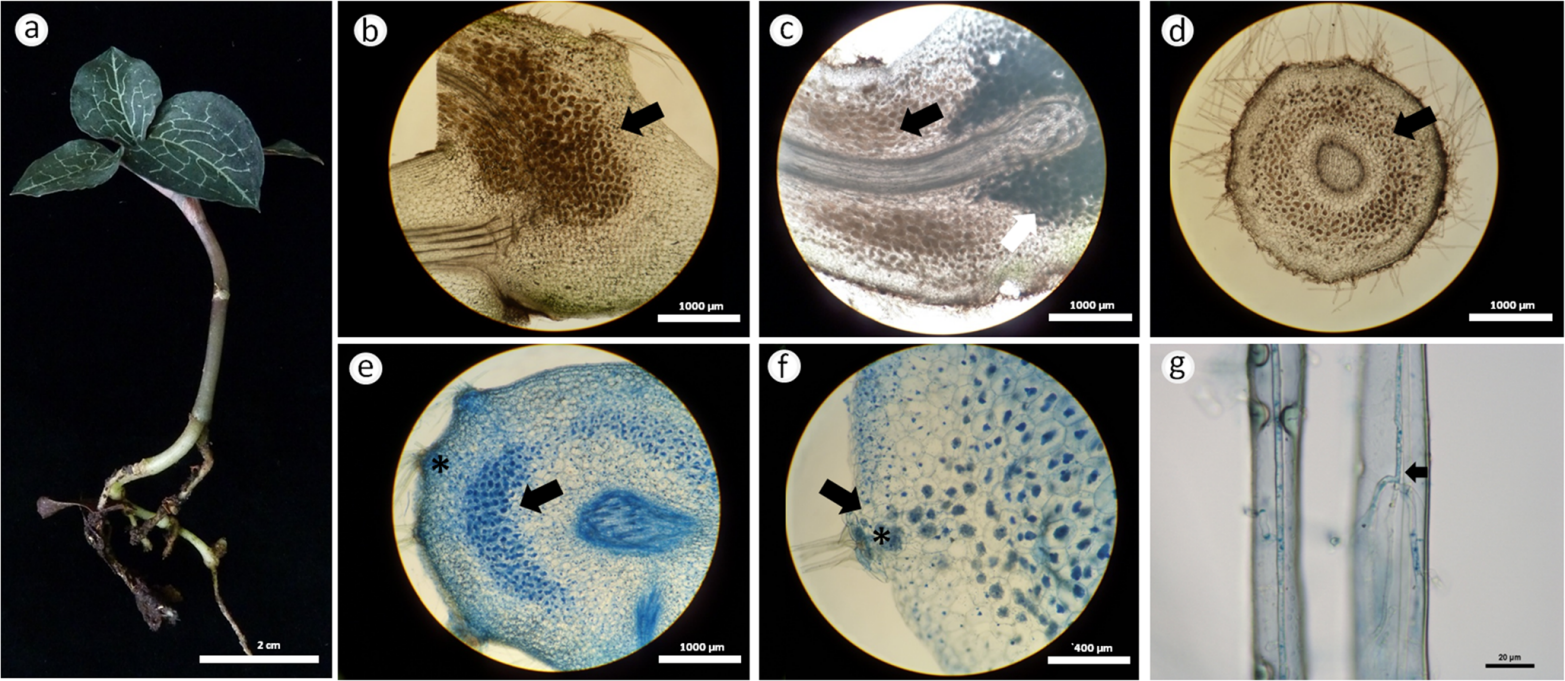
Light microscopic observation of root or rhizome sections of native *A. formosanus* Hayata (**a**) without staining (**b**–**d**) and stained with trypan blue (**e**–**g**). **b**–**c** Longitudinal and transverse sections showed that fungal pelotons (*black arrowheads*) formed at the junction of root and rhizome, and starch granules (*white arrowhead*) accumulated at a nearby vascular bundle in the rhizome. **d** Transverse sections of root close to the rhizome also formed fungal pelotons. **e**–**f** Transverse sections containing pelotons stained with *trypan blue*, pelotons are *dark blue*, and plant tissues are *light blue*. Fungal mycelia entered the cortex through a structure called “hair cushion” (*asterisk*) formed only at the junction of root and rhizome. **g** Fungal mycelia (*small arrow*) can be seen in a single rhizoid of the hair cushion (*asterisk*). *Scale bars* represent 2 cm (**a**), 1,000 μm (**b**–**e**), 400 μm (**f**), and 20 μm (**g**)

All samples of terrestrial *A. formosanus* collected from central and northern regions of Taiwan had fungal pelotons at the junction of the root and rhizome (Fig. 1). To make sure no pertinent information was lost, tissue and peloton isolation (Rasmussen 2002) and direct PCR (Taylor and McCormick 2008) were applied to check and identify the fungal pelotons that existed in cortical cells of *A. formosanus. A. formosanus* samples from a given location often yielded the same cultural type of *Rhizoctonia*, except for the sample from Alishan which contained two different cultural types of *Rhizoctonia* fungi (Table 1). A representative isolate of *Rhizoctonia* was selected from each sample site by using hyphal anastomosis and nuclear staining criteria (Sneh et al. 1991). These isolates showing unique cultural characteristics were subjected to further morphological and molecular characterization. In this study, five of the nine isolates sampled from native *A. formosanus* possessed multinucleate hyphal cells, while the remaining four isolates had binucleate hyphal cells. Among the latter, both isolates ANOF 6 and ANOF G2 were tentatively identified as *Epulorhiza* (teleomorph = *Tulasnella*) based on the colony and hyphal characteristics described by Currah and Zelmer (1992). No teleomorph stage of any isolate of *Rhizoctonia* was observed throughout the study.

### Phylogenetic analyses

Both ITS1/4 and ITS1-OF/ITS4-OF primer sets were used to amplify rDNA-ITS sequences of endomycorrhizal fungi observed in orchid tissues. Although some amplification of fungi in the phylum Ascomycota was observed (i.e., *Fusarium* spp.), *Rhizoctonia* fungi (teleomorphs = *Tulasnella*, *Ceratobasidium*, and *Thanatephorus*) were amplified selectively with primer set ITS1-OF/ITS4-OF which also exhibited more sensitivity than the ITS1/4 primer set in our study. Amplified rDNA-ITS sequences of isolates and uncultured isolates (direct amplification) were sequenced, and the results of BLAST searches were conducted and checked twice. In this study, we found that endomycorrhizal fungi could be easily isolated from the junction of root and rhizome of *A. formosanus* (Fig. 1) and successfully detected at this site by direct amplification with PCR.

**Fig. 2:**
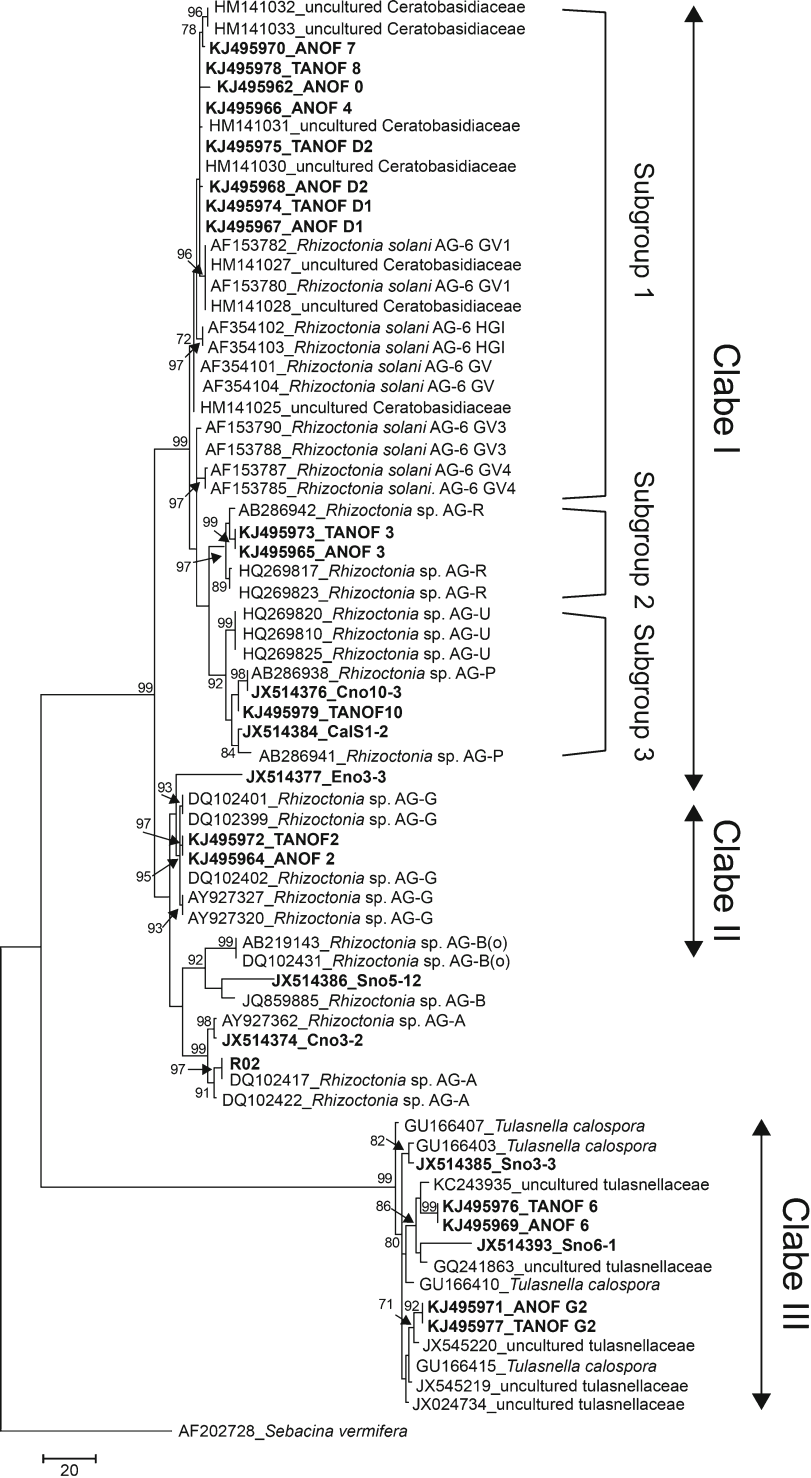
Phylogeny of *Rhizoctonia* isolates from orchids and related fungi based on rDNA-ITS sequences analysis using the maximum parsimony (MP) method. One ITS sequence from *Sebacina vermifera* (AF20272) was defined as an outgroup taxon. *Numbers* at the nodes indicate bootstrap values (1,000 replicates; only values above 70 % are presented) from the corresponding MP tree. The following sequences were direct detection from tissues containing fungal pelotons: TANOF 2, TANOF 3, TANOF D1, TANOF D2, TANOF 6, TANOF G2, TANOF 8, and TANOF 10

The MP analysis divided the endomycorrhizal *Rhizoctonia* from native *A. formosanus* into three major clades (clades I to III) with high bootstrap values. These clades have been further analyzed and categorized into different AGs (Fig. 2), as rDNA-ITS sequences were proven to reflect the classification of AGs within *Rhizoctonia* spp. (Sharon et al. 2006; Sharon et al. 2008). Results of the BLAST searches are shown in Supplemental data 1. Clade I contained isolates from native *A. formosanus* in the northern regions of Taiwan, with the exception of ANOF 7 from the central regions (Alishan). Three sequences of uncultured isolates (TANOF D1, TANOF D2, and TANOF 8) and five sequences of isolates (ANOF 0, ANOF 4, ANOF D1, ANOF D2, and ANOF 7) in clade I shared high rDNA-ITS sequence similarity (97 to 99 % identity) with uncultured isolates of *Rhizoctonia* fungi in the Ceratobasidiaceae sampled from orchid (*Goodyera* spp.) roots (Shefferson et al. 2010). Phylogenetic analysis with maximum parsimony suggested that eight sequences grouped with *Rhizoctonia solani* AG-6 (teleomorph = *Thanatephorus*) (Pope and Carter 2001; Gonzalez et al. 2001) and formed a well-supported subgroup (subgroup 1) within clade I. The identification of isolates of *R. solani* AG-6 was also verified according to the anastomosis group criteria.

Both isolate ANOF 3 and uncultured isolate TANOF 3 in clade I were sampled from different tissues of the same individual plant. They were grouped in subgroup 2 with bootstrap support of greater than 70 %, sharing high sequence similarity (97–99 % identity) with isolates of binucleate *Rhizoctonia* AG-R (teleomorph = *Ceratobasidium*) previously deposited in GenBank (accession numbers HQ269817, HQ269823, and AB286942) (Sharon et al. 2008; Copes et al. 2011). Comparison of the rDNA-ITS sequences suggested that isolate ANOF 3 belongs to AG-R. Uncultured isolate TANOF 10 in clade I formed a well-supported subgroup (subgroup 3) and had sequence similarity to sequences from isolates of binucleate *Rhizoctonia* AG-P (GenBank accession numbers AB286941 and AB286938) with 94 % identity (Sharon et al. 2008) and binucleate *Rhizoctonia* AG-U (accession numbers HQ269810, HQ269820, and HQ269825) with 94 % identity (Copes et al. 2011). Subgroups 2 and 3 were closely related to each other and also related to eight other sequences in subgroup 1 obtained from *A. formosanus* (Fig. 2).

The rDNA-ITS sequences of isolates ANOF 2 and uncultured isolate TANOF 2 were sampled from different tissues of the same individual plant collected in the central region of Taiwan and grouped in clade II, showing 99 % identity to rDNA-ITS sequences from isolates of binucleate *Rhizoctonia* sp. AG-G (accession numbers DQ102399, DQ102401, DQ102402, AY927320, and AY927327) (Manici and Bonora 2007; Sharon et al. 2007). Isolate ANOF 2 was identified as *Rhizoctonia* AG-G (teleomorph = *Ceratobasidium*) based on hyphal anastomosis and rDNA-ITS sequence analyses.

Both isolate ANOF 6 and uncultured isolate TANOF 6 from native *A. formosanus* in the central regions of Taiwan (e.g., Nantou and Alishan) are grouped in clade III, showing 95 % sequence identity to sequences from three isolates of *Tulasnella calospora* previously deposited in GenBank (accession numbers GU166403, GU166407, and GU166410) (Nontachaiyapoom et al. 2010). Furthermore, isolates ANOF G2 and uncultured isolate TANOF G2 also showed high sequence similarity (98 % identity) to isolates of *T. calospora* (accession number GU166403), uncultured members of Tulasnellaceae (accession number JX024734) from *Dactylorhiza* spp. orchids with 99 % identity (Jacquemyn et al. 2012), and uncultured members of Tulasnellaceae (accession numbers JX545220 and JX545219) from *Dendrobium* spp. orchids with 98 % sequence identity (Xing et al. 2013). The sequence comparison supported that these isolates could be identified as *T. calospora*.

### Symbiotic seed germination

**Fig. 3:**
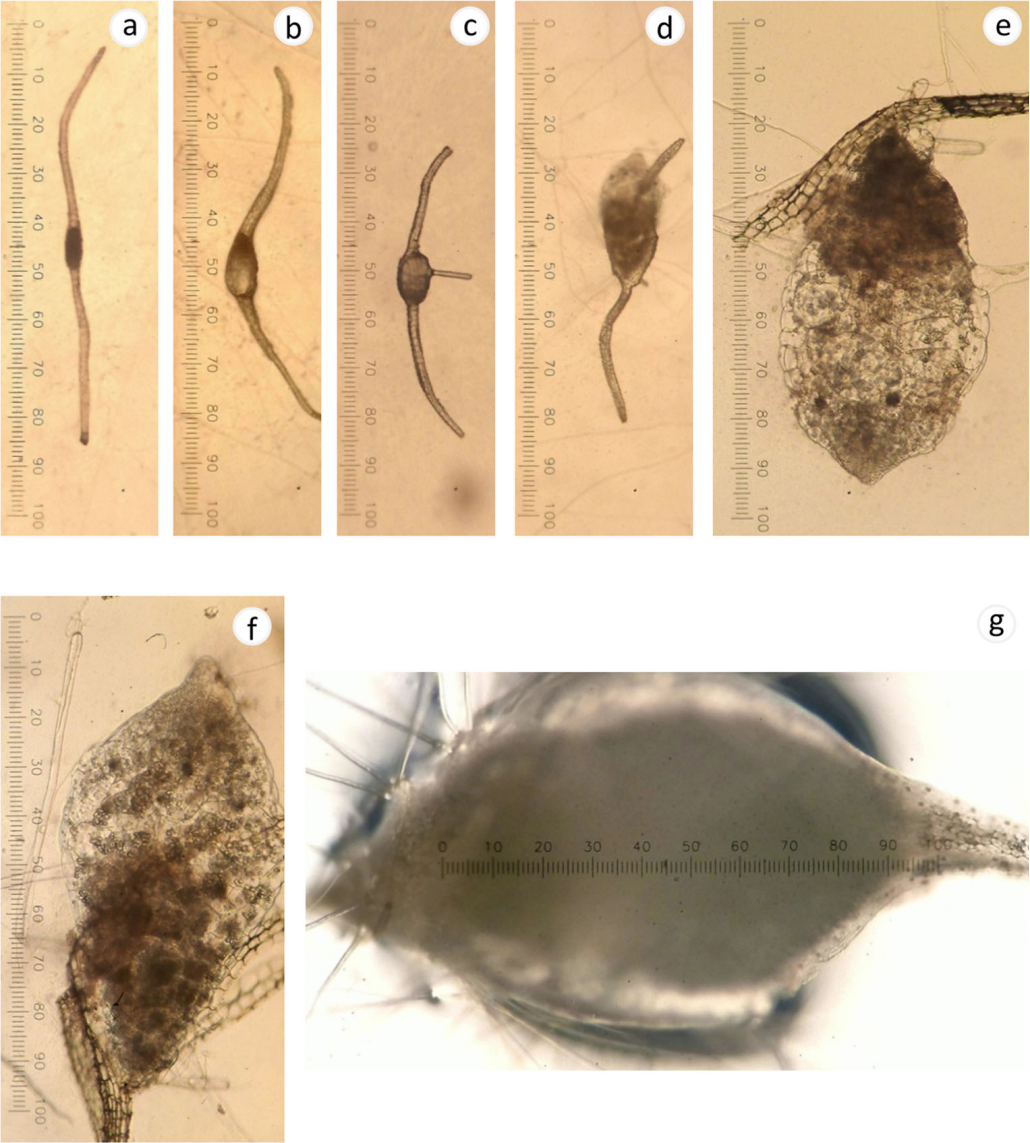
Stages in the development of *A. formosanus* protocorms. **a** Phase 0, an ungerminated seed invested by membranous testa. **b** Phase I, embryo absorbed water and swelled slightly in testa. **c** Phase II, embryo swollen slightly was observed with production of an epidermal hairs that ruptured the testa. **d** Phase III, embryo was large enough to burst the testa and was less than 1,000 μm in length. **e** Phase IV, the length of the embryo was between 1,000 and 2,000 μm. **f** Phase V, the length of the embryo was between 2,000 and 3,000 μm. **g** Phase VI, the embryo was more than 3,000 μm in length. *Scale bars* represent 2,500 μm

**Fig. 4:**
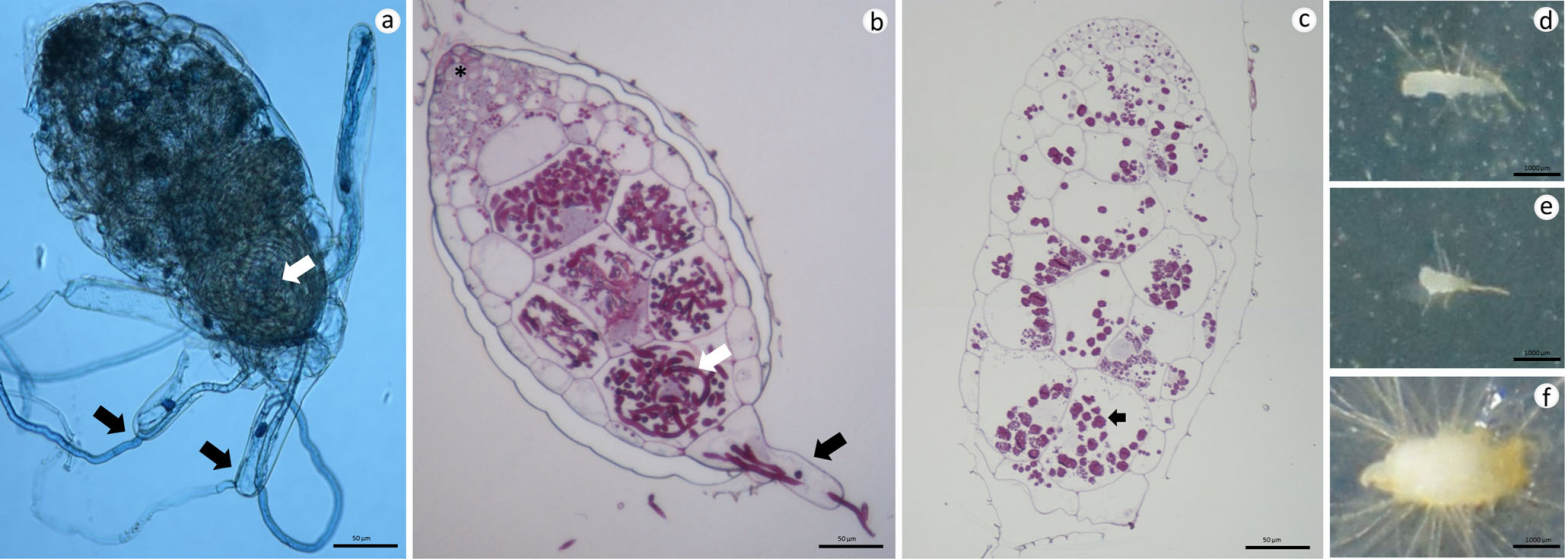
Structures of *A. formosanus* protocorm after 10 days of culture (**a**–**c**) and 60 days of culture (**d**–**f**) with fungus (symbiotic) and noninoculated (asymbiotic) treatments. **a** Direct observation by light microscopy. **b**, **c** Sections were stained with periodic acid-Schiff (PAS) reagent. Carbohydrates stained purplish red and nuclei lightly stained. **a**, **b** Fungal mycelia have penetrated into basal cell(s) or rhizoid(s) (*black arrowheads*) and then into the inner cortical parenchymal cells and have formed fungal pelotons (*white arrowheads*). The meristematic region was not colonized (*asterisk*). **c** Non-inoculated treatment is the same size as the inoculated treatment but contains many granules (*small arrowhead*), which are almost absent in fungal treatments. **d**–**f** Protocorms growing on 1/2 strength MS (**d**) and 0.25 % OMA (**e**) without fungal inoculation were smaller and flatter than those in fungal treatments on 0.25 % OMA (**f**). *Scale bar* represents 50 μm (**a**–**c**) and 1,000 μm (**d**–**f**)

The emergence of a swollen embryo from the seed coat was used as the criterion to define seed germination (phase III, Fig. 3). The embryo began to swell when hyphae invaded the basal cells (Fig. 4a). Hyphae penetrated into the inner cortical parenchyma and subepidermal parenchyma cells and formed pelotons (Fig. 4b). The meristematic region was not colonized (Fig. 4b).

**Fig. 5:**
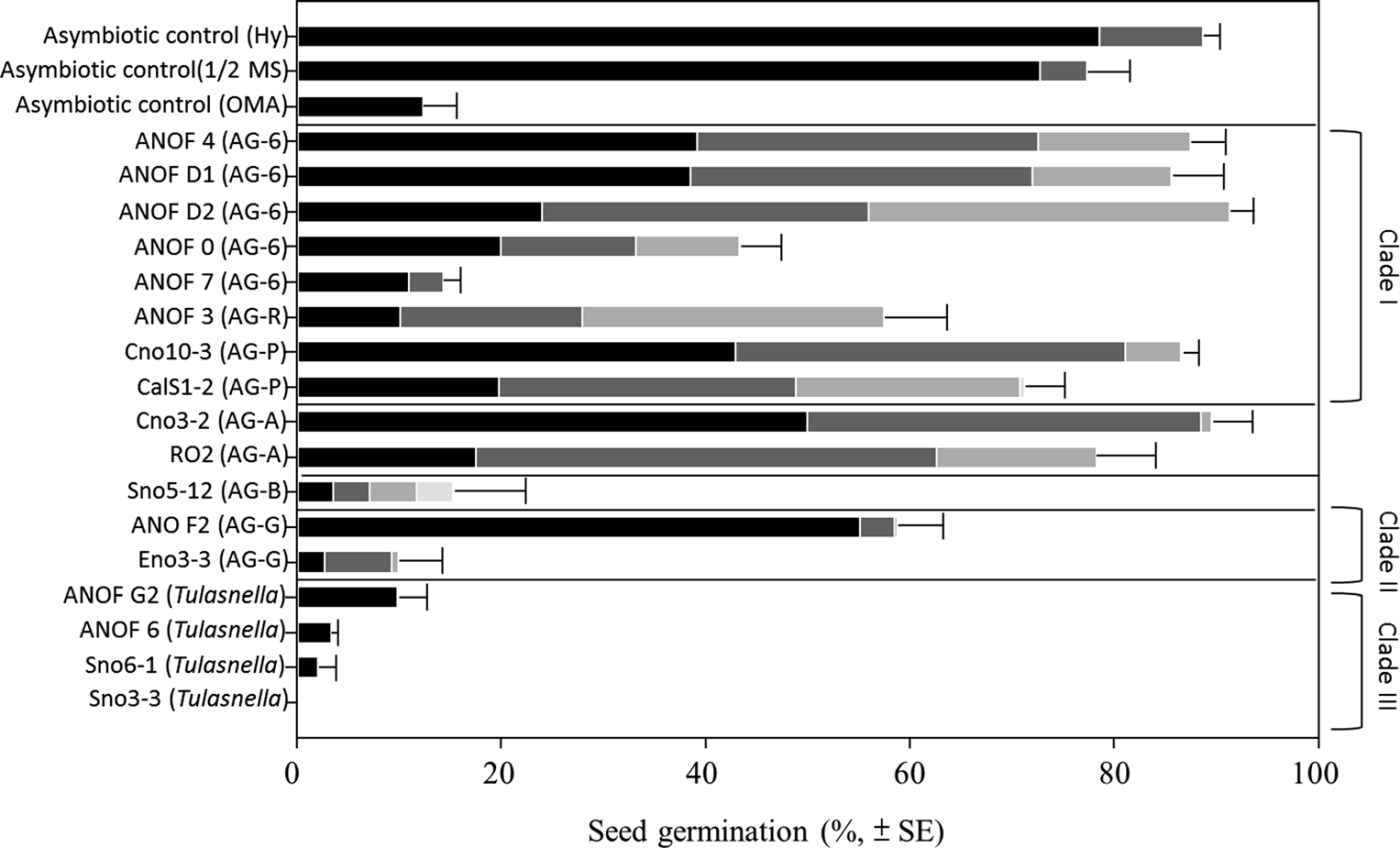
Asymbiotic and symbiotic seed germination of *A. formosanus* Hayata, recorded 30 days after sowing seeds on 0.25 % oatmeal agar (OMA), 1/2 strength Murashige-Skoog (MS) medium, and Hyponex medium for asymbiotic control and on 0.25 % OMA for symbiotic treatments. Phase III was used to define whether a seed had germinated. Percentage of germinated seeds was presented as () in phase III, () in phase IV, () in phase V, and () in phase VI

Germination for the asymbiotic seeds was 89 % on Hy medium, 75 % on 1/2 strength MS, and 13 % on 0.25 % OMA. However, most of the germinated seeds only grew to phase III (Fig. 5) after 30 days of incubation. Seeds sown on 0.25 % OMA without fungal inoculation remained in phase III after 60 days of culture (Fig. 4e). For the remaining two asymbiotic treatments (Hy medium and 1/2 strength MS), growth of protocorms into stages IV–V was observed after 60 days of culture (Fig. 4d), but they were smaller and flatter in width than those in the symbiotic treatment (0.25 % OMA) (Fig. 4f). Fungal pelotons formed in germinated *A. formosanus* seeds, indicating that germination was associated with the presence of the fungus (Figs. 4 and 5). Increased germination was observed with isolates found in clade I and ranged from 44 to 91 % compared to asymbiotic treatment (13 %) in 0.25 % OMA and promoted growth of protocorms to phases IV and V. However, isolate ANOF 7 in clade I from central regions of Taiwan (Alishan) did not increase seed germination (14 %).

Both isolates ANOF G2 and ANOF 6 (teleomorph = *Tulasnella*) in clade III sampled from *A. formosanus* in central regions of Taiwan (Nantou and Alishan) did not increase seed germination (3–10 %) in 0.25 % OMA (Fig. 5). Isolates Sno3-1 and Sno6-1, which were closely related to isolates ANOF G2 and ANOF 6 in clade III, were sampled from the northern region (Sanxia) and also did not increase seed germination (0–2 %). Results suggested that isolates of *T. calospora* can form fungal pelotons in cortical cells of native *A. formosanus* growing in central regions but did not increase seed germination under our laboratory-based assay conditions. Isolate ANOF 2 in clade II, which was also sampled from *A. formosanus* in central Taiwan, increased seed germination (59 %); however, almost all the germinated seeds grew slowly into phase III after 30 days of co-culture compared to the other treatments with fungi in clade I (Fig. 5).

### Colonization of endomycorrhizal fungi

**Fig. 6:**
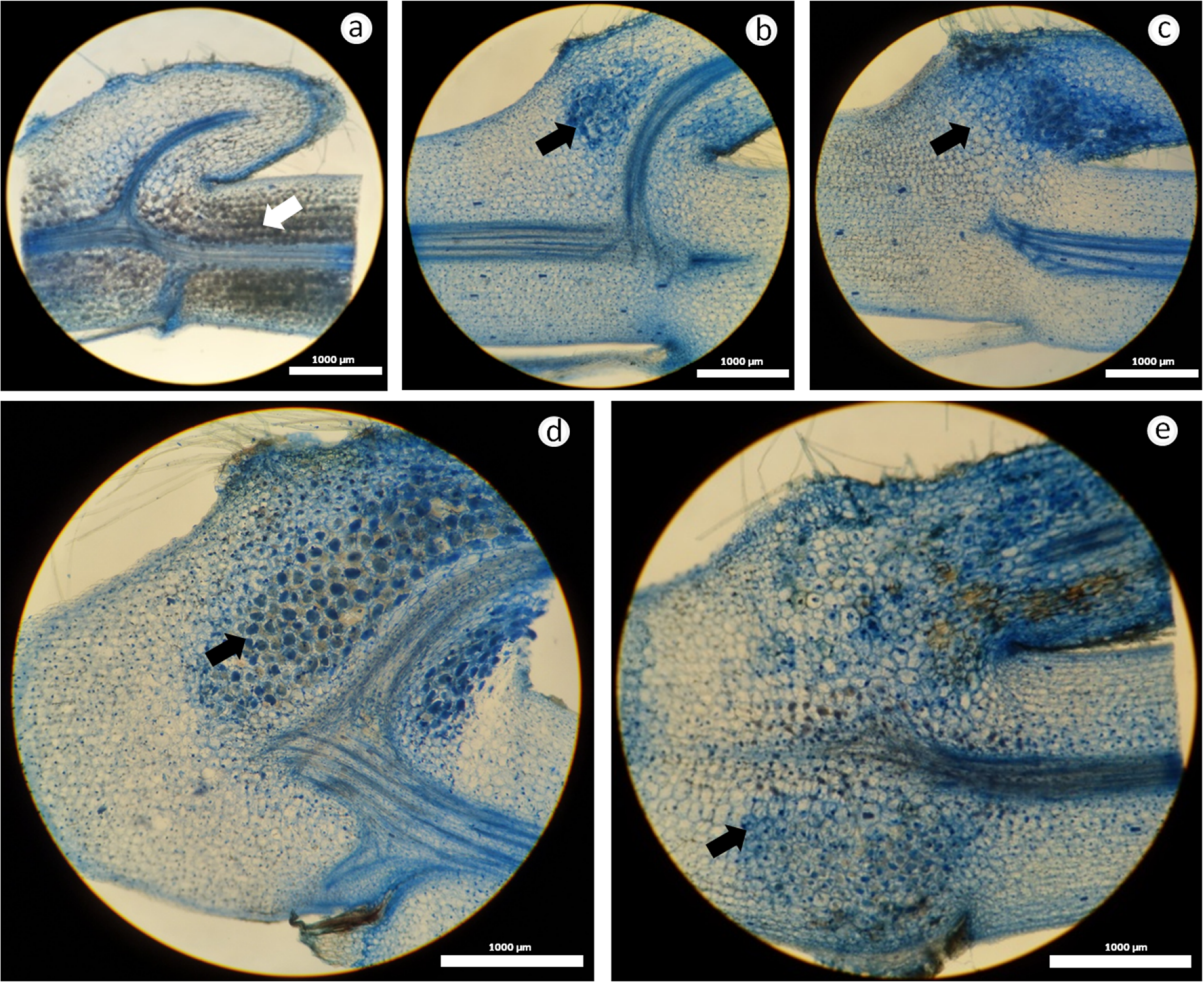
Hand sections of symbiotic and asymbiotic treatments at each node of *A. formosanus* showing different levels of colonization after 120 days of culture. **a** 0 = fungal pelotons were absent and *white arrowhead* stands for starch granules. **b** 1 = cortex was slightly colonized and formed a few pelotons (*black arrowheads*) at the junction of rhizome and branching root. **c** 2 = more fungal pelotons have formed at the upper and lower site in the branching root. **d** 3 = the basal part of the branching root (1–3 mm) was full of fungal pelotons, which have spread to rhizome. **e** 4 = branching root and rhizome were full of fungal pelotons. *Scale bars* represent 1,000 μm

In general, tissue-cultured seedling inoculated with any isolate of *Rhizoctonia* increased the fresh plant weight compared to asymbiotic treatment after 120 days of co-culture in vitro (Table 2). However, plant growth was affected by different isolates of *Rhizoctonia* fungi. By sectioning each part of rhizome and roots, the results showed that plantlets had massive pelotons at the junction of root and rhizome (Fig. 6). Calculating the relative treatment effects for a 0-to-4 scale of fungal colonization suggested that isolates in clade I significantly promote the growth of seedlings with a relatively low level of colonization (${\widehat{p}}_i = 0.30\hbox{--} 0.47$) compared to the other isolates in clades II (${\widehat{p}}_i = 0.63\hbox{--} 0.82$) and III (${\widehat{p}}_i = 0.63\hbox{--} 0.75$) with 95 % confidence interval. Our data also indicated that the increase due to the treatment effects on fungal colonization was negatively correlated (*r* = −0.8801) with fresh weight of tissue-cultured seedling (Supplemental data II).

**Table 2:** Plant weight, median, and relative treatment effects (${\widehat{p}}_i$) with 95 % confidence intervals (CI) for the degree of colonization on tissue-cultured seedlings of *A. formosanus* caused by endomycorrhizal isolates of *Rhizoctonia* after 120 days of co-culture

Isolate	AG^a^	Clade^b^	Weight (mg)^c^	Relative treatment effect (${\widehat{p}}_i$) for colonization rating^d^		
				Median	${\widehat{p}}_i$	95 % CI for ${\widehat{p}}_i$
Non-inoculated	–	–	363.8 g	0	0.069	(0.056, 0.088)
*Thanatephorus*						
ANOF 0	AG-6	I	590.6 b–d	2	0.432	(0.309, 0.564)
ANOF 4	AG-6	I	688.5 ab	1	0.337	(0.247, 0.443)
ANOF D2	AG-6	I	645.8 a–c	1	0.300	(0.239, 0.371)
*Ceratobasidium*						
ANOF 3	AG-R	I	571.9 b–e	1.5	0.466	(0.347, 0.590)
Cno10-3	AG-P	I	670.9 a–c	2	0.452	(0.371, 0.535)
CalS1-1	AG-P	I	717.2 ab	1	0.316	(0.251, 0.392)
Cno3-2	AG-A	–	756.5 a	1	0.375	(0.313, 0.441)
R02	AG-A	–	775.3 a	1	0.362	(0.294, 0.437)
Sno5-12	AG-B	–	436.1 fg	4	0.871	(0.825, 0.904)
Eno3-3	AG-G	II	517.9 d–f	3.5	0.822	(0.748, 0.874)
ANOF 2	AG-G	II	527.5 c–f	3	0.633	(0.500, 0.747)
*Tulasnella*						
ANOF 6	–	III	458.3 e–g	3	0.681	(0.589, 0.759)
ANOF G2	–	III	510.6 d–g	3	0.633	(0.547, 0.709)
Sno3-3	–	III	424.7 fg	3	0.752	(0.643, 0.832)

^a^Isolates were identified as different anastomosis groups (AGs) based on AG determination and rDNA-ITS sequences except for the isolates of *Tulasnella*

^b^The “ANOF” designation represents isolates sampled from native *A. formosanus* Hayata and classified into clade I, II, or III according to phylogenetic analysis of ITS rDNA sequences. The other isolates were sampled from a variety of green orchids described in the “Materials and methods”

^c^Plant fresh weight was determined as described in the “Materials and methods.” Values followed by the same letter are not significantly different according to one-way analysis of variance (ANOVA) followed by Tukey’s multiple range test (*P* = 0.05)

^d^Relative treatment effects were calculated by performing one-way analyses using the nonparametric method for ordinal data described by Shah and Madden (2004). The values of ${\widehat{p}}_i$ between two accessions are significantly different from each other if 95 % CI for each ${\widehat{p}}_i$ does not overlap

– Represents a non-determined element

## Discussion

### Characterization and identification of endomycorrhizal fungi

The endomycorrhizal fungi sampled from green orchids has been recognized as belonging to the form genus of *Rhizoctonia* that include species of *Thanatephorus*, *Ceratobasidium*, *Tulasnella*, and *Sebacina* (Sneh et al. 1991; Roberts 1999; Dearnaley 2007; Smith and Read 2008). All isolates from fungal pelotons in native *A. formosanus* belonged to the form genus *Rhizoctonia*, including *Thanatephours*, *Ceratobasidium*, and *Tulasnella*. In our study, none of the isolates from *A. formosanus* were identified as *Sebacina*, which can form endomycorrhizae with several green orchids in Australia, especially in fully mycoheterotrophic orchids (McKendrick et al. 2002; Selosse et al. 2002; Taylor et al. 2003).

The method of AG determination is a commonly used procedure to characterize and identify isolates of *Ceratobasidium* and *Thanatephorus* since the sexual stage of these fungi is often challenging to induce in the laboratory (Sneh et al. 1991; Carling et al. 1999). Many AGs have been found on orchids and increased our understanding of orchid mycorrhizal symbionts (Ramsay et al. 1987). Currently, the multinucleate *R. solani* (teleomorph = *Thanatephorus*) contains 14 AGs (González García et al. 2006), while the binucleate *Rhizoctonia* spp. (teleomorph = *Ceratobasidium*) contains at least 16 AGs (Sharon et al. 2008). Three AGs of *Thanatephorus* (AG-5, AG-6, and AG-12) and four AGs of *Ceratobasidium* (AG-A, AG-C, AG-E, and AG-I) have been reported to form mycorrhizal associations with orchids in nature (Sneh et al. 1991; Carling et al. 1999; Pope and Carter 2001). Fungi in the same AG may possess similar morphological characteristics or specialized host ranges (Sneh et al. 1991; Keijer et al. 1997). With molecular analysis and AG determination, isolates from native *A. formosanus* were identified as multinucleate *R. solani* AG-6, binucleate *Rhizoctonia* sp. AG-G, AG-P, and AG-R, and *Rhizoctonia repens* (teleomorph = *T. calospora*) (Fig. 2). Sharon et al. (2008) have suggested that AG-P are closely related with AG-U based on rDNA-ITS sequence similar and may represent the same anastomosis group. However, additional analyses with multiple genetic loci are needed to better substantiate these findings.

Previously on the studies on phylogenetic relationships of *Rhizoctonia* fungi, the genus *Ceratobasidium* was paraphyletic and *R. solani* AG-6 often grouped with isolates of *Ceratobasidium* (Gonzalez et al. 2001; Moncalvo et al. 2006). These observations were similar to those reported by Sharon et al. (2008). *R. solani* AG-6 was the predominant mycorrhizal fungus isolated from fungal pelotons of *A. formosanus* in the northern regions of Taiwan and represented 67 % of the sample. In this study, we found that *Ceratobasidium* (i.e., AG-R and AG-P) was grouped in clade I with AG-6 and all of isolates from northern regions except isolate ANOF 7 from central regions. The results suggest that isolates in clade I represent the predominant endomycorrhizal symbionts associated with *A. formosanus* in nature (Fig. 2). Interestingly, some isolates of AG-P and AG-R were previously recorded as plant pathogenic fungi (Sharon et al. 2008; Yang et al. 2008), but certain isolates were also used for protecting plants against pathogenic fungi (Sneh and Ichielevich-Auster 1998; Jabaji-Hare and Neate 2005). A recent study reported from Veldre et al. (2013) suggested that the sequences of *Rhizoctonia* fungi derived from soil (representing putative saprobes) and orchid mycorrhizae clustered together, but remained distinct from pathogens, and that autotrophic orchids generally form root symbioses with available Ceratobasidiaceae isolates in soil.

Isolates ANOF 2 (AG-G) and both isolates of ANOF 6 and ANOF D2 (*Tulasnella*) from the central regions of Taiwan were distributed in clades II and III, respectively. Isolate ANOF 7 (AG-6) in clade I was also sampled from *A. formosanus* in central regions of Taiwan. It cannot be stated with certainty that the diversity of *Rhizoctonia* spp. in *A. formosanus* from central regions was different from northern regions due to our limited sample size as native *A. formosanus* is challenging to collect in central Taiwan. However, the environment for plant growth (i.e., substrate and seasonal changes) has been reported to influence the fungal preference and diversity of orchid-associated fungi (Porras-Alfaro and Bayman 2007; Selosse and Roy 2009; Otero et al. 2011).

### Symbiotic seed germination

The method of symbiotic seed germination demonstrates relationships between seeds and endomycorrhizal fungi. *A. formosanus* embryo formed fungal pelotons at the mycorrhizome after 10 days of culture (Fig. 4). The disappearance of starch grains in amyloplasts has frequently been reported by several researchers when they observed that a mycorrhizal fungus invaded cells of orchid roots and protocorms (Richardson et al. 1992; Uetake et al. 1992).

Analyzing the data of seed germination (Fig. 5) with the result of phylogenetic analysis (Fig. 2) suggested that isolates sampled from *A. formosanus* in northern regions of Taiwan in clade I (i.e., subgroups 1 to 3), except for isolate ANOF 7, effectively increase seed germination and promote the growth of protocorm into phases IV–V. It is worth mentioning that both isolates CalS1-2 and Cno10-3 were sampled previously from other green orchids. Our data suggests that *A. formosanus* seeds possess mycorrhizal preference with isolates in clade I under in vitro experimental conditions. Additionally, isolates of *Ceratobasidium* AG-A in northern regions of Taiwan also showed symbiotic ability with *A. formosanus* seeds similar to the responses observed for fungi in clade I. The ability of isolates of *Ceratobasidium* to stimulate orchid seed germination and seedling growth was predicted by phylogenetic relationships (Otero et al. 2005), suggesting a genetic basis for mycorrhizal symbiosis. Nevertheless, isolate ANOF 7 in clade I (subgroup 1) from the central region of Taiwan (Alishan) did not increase seed germination, implying that isolates from different geographic regions varied in symbiotic ability within the population of *A. formosanus*. However, additional studies are needed to ascertain the genetic diversity of isolates sampled from different geographic locations to increase the predictability of phylogenetic-based methods for identifying potential endomycorrhizal symbionts.

Tulasnelloid fungi especially species of *Tulasnella* are well-studied endomycorrhizal fungus associated with many green orchids and promote seed germination (Suarez et al. 2006). Our results suggested that isolates of AG-G in clade II and *Tulasnella* species in clade III sampled from *A. formosanus* in central regions and other green orchids in northern regions did not increase germination of *A. formosanus* seeds and promote increased plant development (Fig. 5). Although seeds inoculated with ANOF 2 expressed a moderate increase in seed germination, only a few seeds grew to phase IV after 60 days of inoculation and exhibited limited growth. Therefore, the mycorrhizal specificity of *A. formosanus* was also possibly associated with the geographic origin of the orchid seeds and/or *Rhizoctonia* fungus. This difference in the mycorrhizal specificity of *A. formosanus* may also be explained by the fact that seeds instead of adult plants were utilized for the symbiotic experiments, i.e., the difference in specificity was due to physiological differences in the developmental stage of the orchid (Masuhara et al. 1993). Although our data suggest that mycorrhizal specificity of *A. formosanus* seeds with *Rhizoctonia* fungi in clade I may occur, further studies of seed germination in the same or other geographic locations under in situ conditions are warranted to better document and substantiate this observation.

### Colonization of endomycorrhizal fungi

Mycorrhizal specificity in relation to orchid diversity has been studied in field conditions (Masuhara and Katsuya 1994; McKendrick et al. 2002; McCormick et al. 2004). Because the populations of native *A. formosanus* have greatly decreased the occurrence of this species in central and northern Taiwan, we conducted a synthesis study by using tissue-cultured seedlings when a pure culture can be obtained from fungal pelotons to further demonstrate functionality and fungal–plant interactions. As mentioned above, isolates in clade II and clade III expressed low or no ability to increase seed germination in vitro. However, when using tissue-cultured seedlings, all isolates we studied formed fungal pelotons at the junction of roots and rhizome. The results showed that mycorrhizal specificity of *A. formosanus* seeds was different from tissue-cultured seedlings.

*A. formosanus* will increase the efficacy of photosynthesis by inoculating endomycorrhizal *Rhizoctonia* fungi (Cheng and Chang 2009). Indeed, when a protocorm grows into a seedling with a differentiated green shoot, the presence of chlorophyll may not be sufficient to enable full autotrophy (Smith and Read 2008). It has been demonstrated previously that the pathway for carbon transfer from the fungus to plant is retained into adulthood in autotrophic species of orchids and suggests that green orchids are partially mycoheterotrophic (“mixotrophic”) (Gebauer and Meyer 2003; Julou et al. 2005; Cameron et al. 2006). However, it is unlikely that endomycorrhizal fungi derive other nutritional benefits from their association with the orchid (Leake 2005).

In general, all isolates examined in this study significantly increased the fresh weight of tissue-cultured seedlings compared to the asymbiotic treatment (Table 2), irrespective of their genetic relatedness, geographic origin, and ability to promote seed germination of *A. formosanus*. However, we found that fungal colonization was negatively correlated with fresh weight of plantlets (*r* = −0.8801) (Supplemental II). Isolates in clade I (i.e., subgroups 1 to 3) increased seed germination and significantly promoted the growth of tissue-cultured seedlings with low relative treatment effects ranging from 0.30 to 0.47. Although the fresh weights of plantlets with high relative treatment effects caused by isolate Eno3-3 (${\widehat{p}}_i = 0.82$) sampled from orchid *G. procera* and isolate Sno5-12 (${\widehat{p}}_i = 0.87$) from *C. paniculatum* were greatly decreased, all plants were alive and had greener leaves than those in asymbiotic treatments. Endomycorrhizal *Rhizoctonia* fungi might be considered to be parasitic on orchids when the net cost of the symbiosis exceeds any net benefits, and parasitism can be induced developmentally, environmentally, or possibly genotypically. Therefore mycorrhizal symbiosis was described as a mutualism–parasitism continuum (Johnson et al. 1997; Newton et al. 2010).

The colonization by isolates in clade II (${\widehat{p}}_i = 0.63\hbox{--} 0.82$) and clade III (${\widehat{p}}_i = 0.63\hbox{--} 0.75$) was similar and limited the growth of *A. formosanus* plantlets, which did not decay and continued growing with a slight brown coloration at the base of the rhizome. This basal part of rhizome had cortical cells that were completely colonized with fungal pelotons. Fungitoxic phytoalexins, cell wall-degrading enzymes, and species of reactive oxygen can occur in tubers or rhizomes and are hypothesized to regulate or prevent fungal colonization (Stoessl and Arditti 1984; Beyrle et al. 1995). However, their role in the process of peloton digestion and formation is not clear (Smith and Read 2008). Paduano et al. (2011) demonstrated that pectin is exclusively found in the interface formed around *Ceratobasidium* sp. but not *Russula* sp. and that the native green orchid *Limodorum abortivum* expressed different cellular responses to the mycorrhizal fungi. Using tissue-cultured seedlings as symbiotic materials for further investigation of the fungal–plant interface associated with different levels of colonization under laboratory condition may potentially uncover important key interactions for partially mycoheterotrophic *A. formosanus* in regulating fungal colonization, growth, and development.

## Conclusion

The population of native *A. formosanus* is associated with endomycorrhizal *Rhizoctonia* fungi, especially *R. solani* AG-6. These isolates were divided into clades I to III and differed in their ability to colonize orchid seeds and tissue-cultured seedlings in vitro assays. Our data suggest that the majority of isolates sampled from native *A. formosanus* in northern regions of Taiwan in clade I have ability to increase seed germination successfully and also effectively promote the growth of tissue-cultured seedlings with a low relative treatment effects for the degree of colonization. By comparison with isolates in clade I, an increased value of the relative treatment effect exhibited by isolates in clades II and III reduced the growth of *A. formosanus*. Our findings will be potentially useful for medicinal orchid production and conservation of *A. formosanus* in diverse and ecologically sensitive native habitats.

## Electronic supplementary material

Below is the link to the electronic supplementary material.

ESM 1 (DOCX 47 kb)

ESM 2 (DOCX 79 kb)
