# Supplementary material for: Characterization and colonization of endomycorrhizal Rhizoctonia fungi in the medicinal herb Anoectochilus formosanus (Orchidaceae)
Source: Mycorrhiza. 2015 Jan 11;25(6):431–45. doi: 10.1007/s00572-014-0616-1 (PMC4512280; doi:10.1007/s00572-014-0616-1)
Supplement: Supplementary file 1 — (DOCX 47 kb) [file 572_2014_616_MOESM1_ESM.docx]

**Supplemental data I** Isolates of endomycorrhizal *Rhizoctonia* fungi from fungal pelotons in terrestrial *Anoectochilus formosanus* Hayata

| Isolate /  Accession No. |  | Highest Blast hit based^a^ |  | % Similarity |  | Reference |
| --- | --- | --- | --- | --- | --- | --- |
| ANOF 0 / KJ495962 |  | AF153780 / isolate OT2-1  *Rhizoctonia solani* |  | 98% |  | Pope and Carter, 2001 |
|  |  | AF354102 / isolate 70Rs  *Rhizoctonia solani* AG6-HGI |  | 97% |  | Gonzalez et al,  2001 |
| ANOF 4 / KJ495966 |  | AF354102 / isolate 70Rs  *Rhizoctonia solani* AG6-HGI |  | 97% |  | Gonzalez et al,  2001 |
|  |  | AF354104 / isolate 75Rs  *Rhizoctonia solani* AG6-GV |  | 97% |  | Gonzalez et al,  2001 |
| ANOF D1 / KJ495967 |  | AF354104 / isolate 75Rs  *Rhizoctonia solani* AG6-GV |  | 97% |  | Gonzalez et al,  2001 |
|  |  | AF354101 / isolate HN1-1  *Rhizoctonia solani* AG6-GV |  | 97% |  | Gonzalez et al,  2001 |
| TANOF D1 / KJ495974 |  | AF354104 / isolate 75Rs  *Rhizoctonia solani* AG6-GV |  | 97% |  | Gonzalez et al,  2001 |
|  |  | AF354102 / isolate 70Rs  *Rhizoctonia solani* AG6-HGI |  | 97% |  | Gonzalez et al,  2001 |
| ANOF D2 / KJ495968 |  | AF354104 / isolate 75Rs  *Rhizoctonia solani* AG6-GV |  | 97% |  | Gonzalez et al,  2001 |
|  |  | AF354102 / isolate 70Rs  *Rhizoctonia solani* AG6-HGI |  | 97% |  | Gonzalez et al,  2001 |
| TANOF D2 / KJ495975 |  | AF354104 / isolate 75Rs  *Rhizoctonia solani* AG6-GV |  | 97% |  | Gonzalez et al,  2001 |
|  |  | AF354102 / isolate 70Rs  *Rhizoctonia solani* AG6-HGI |  | 97% |  | Gonzalez et al,  2001 |
| ANOF 2 / KJ495964 |  | DQ102402 / isolate Str14  *Ceratobasidium* sp. AG-G |  | 99% |  | Sharon et al,  2007 |
|  |  | AY927320 / isolate R13  *Rhizoctoni*a sp. AG-G |  | 99% |  | Manici and Bonora,  2007 |
| TANOF 2 / KJ495972 |  | DQ102402 / isolate Str14  *Ceratobasidium* sp. AG-G |  | 99% |  | Sharon et al,  2007 |
|  |  | AY927320 / isolate R13  *Rhizoctonia* sp. AG-G |  | 99% |  | Manici and Bonora, 2007 |
| ANOF 3 / KJ495965 |  | AB286942 / isolate: X4-3  *Ceratobasidium* sp. AG-R |  | 99% |  | Sharon et al,  2008 |
|  |  | HQ269823 / isolate RhMY074WAz3  *Ceratobasidium* sp. AG-R |  | 98% |  | Copes et al,  2011 |
| TANOF 3 / KJ495973 |  | AB286942 / isolate: X4-3  *Ceratobasidium* sp. AG-R |  | 99% |  | Sharon et al,  2008 |
|  |  | HQ269823 / isolate RhMY074WAz3  *Ceratobasidium* sp. AG-R |  | 98% |  | Copes et al,  2011 |
| ANOF 6 / KJ495969 |  | GU166403 / isolate Ps-KT-0-1  *Tulasnella calospora* |  | 95% |  | Nontachaiyapoom  et al. 2010 |
|  |  | GU166407 / isolate Pch-QS-0-1  *Tulasnella calospora* |  | 95% |  | Nontachaiyapoom  et al. 2010 |
|  |  | GU166410 / isolate Da-KP-0-1  *Tulasnella calospora* |  | 95% |  | Nontachaiyapoom  et al. 2010 |
| TANOF 6 / KJ495976 |  | GU166403 / isolate Ps-KT-0-1  *Tulasnella calospora* |  | 95% |  | Nontachaiyapoom  et al. 2010 |
|  |  | GU166407 / isolate Pch-QS-0-1  *Tulasnella calospora* |  | 95% |  | Nontachaiyapoom  et al. 2010 |
|  |  | GU166410 / isolate Da-KP-0-1  *Tulasnella calospora* |  | 95% |  | Nontachaiyapoom  et al. 2010 |
| ANOF 7 / KJ495970 |  | AF354104 / isolate 75Rs  *Rhizoctonia solani* AG6-GV |  | 97% |  | Gonzalez et al,  2001 |
|  |  | AF354101 / isolate HN1-1  *Rhizoctonia solani* AG6-GV |  | 97% |  | Gonzalez et al,  2001 |
| ANOF G2 / KJ495971 |  | JX024734 / isolate Di_Aga_3D3  Uncultured Tulasnellaceae clone |  | 99% |  | Jacquemyn et al. 2012 |
|  |  | GU166403 / isolate Ps-KT-0-1  *Tulasnella calospora* |  | 98% |  | Nontachaiyapoom  et al. 2010 |
|  |  | JX545220 / DOf-XL17  Uncultured Tulasnellaceae clone |  | 98% |  | Xing et al.  2013 |
| TANOF G2 / KJ495977 |  | JX024734 / isolate Di_Aga_3D3  Uncultured Tulasnellaceae clone |  | 99% |  | Jacquemyn et al. 2012 |
|  |  | GU166403 / isolate Ps-KT-0-1  *Tulasnella calospora* |  | 98% |  | Nontachaiyapoom  et al. 2010 |
|  |  | JX545220 / DOf-XL17  Uncultured Tulasnellaceae clone |  | 98% |  | Xing et al.  2013 |
| TANOF 8 / KJ495978 |  | AF354102 / isolate 70Rs  *Rhizoctonia solani* AG6-HGI |  | 97% |  | Gonzalez et al,  2001 |
|  |  | AF354104 / isolate 75Rs  *Rhizoctonia solani* AG6-GV |  | 97% |  | Gonzalez et al,  2001 |
| TANOF 10 / KJ495979 |  | AB286941 / isolate: C-578  *Ceratobasidium* sp. AG-P |  | 94% |  | Sharon et al,  2008 |
|  |  | AB286938/ isolate: C-584  *Ceratobasidium* sp. AG-P |  | 94% |  | Sharon et al,  2008 |
|  |  | HQ269810 / isolate RhFB078WDz2  *Ceratobasidium* sp. AG-U |  | 94% |  | Cpoes et al,  2011 |
|  |  | HQ269820 / isolate RhMY072WAz3  *Ceratobasidium* sp. AG-U |  | 94% |  | Cpoes et al,  2011 |

^a^ The rDNA-ITS sequences were obtained from GeneBank and the reference sequence with highest blast hit was selected based on a known anastomosis group or a known teleomorphic stage of *Rhizoctonia* fungus
