# Supplementary material for: Characterization and colonization of endomycorrhizal Rhizoctonia fungi in the medicinal herb Anoectochilus formosanus (Orchidaceae)
Source: Mycorrhiza. 2015 Jan 11;25(6):431–45. doi: 10.1007/s00572-014-0616-1 (PMC4512280; doi:10.1007/s00572-014-0616-1)
Supplement: Supplementary file 2 — (DOCX 79 kb) [file 572_2014_616_MOESM2_ESM.docx]

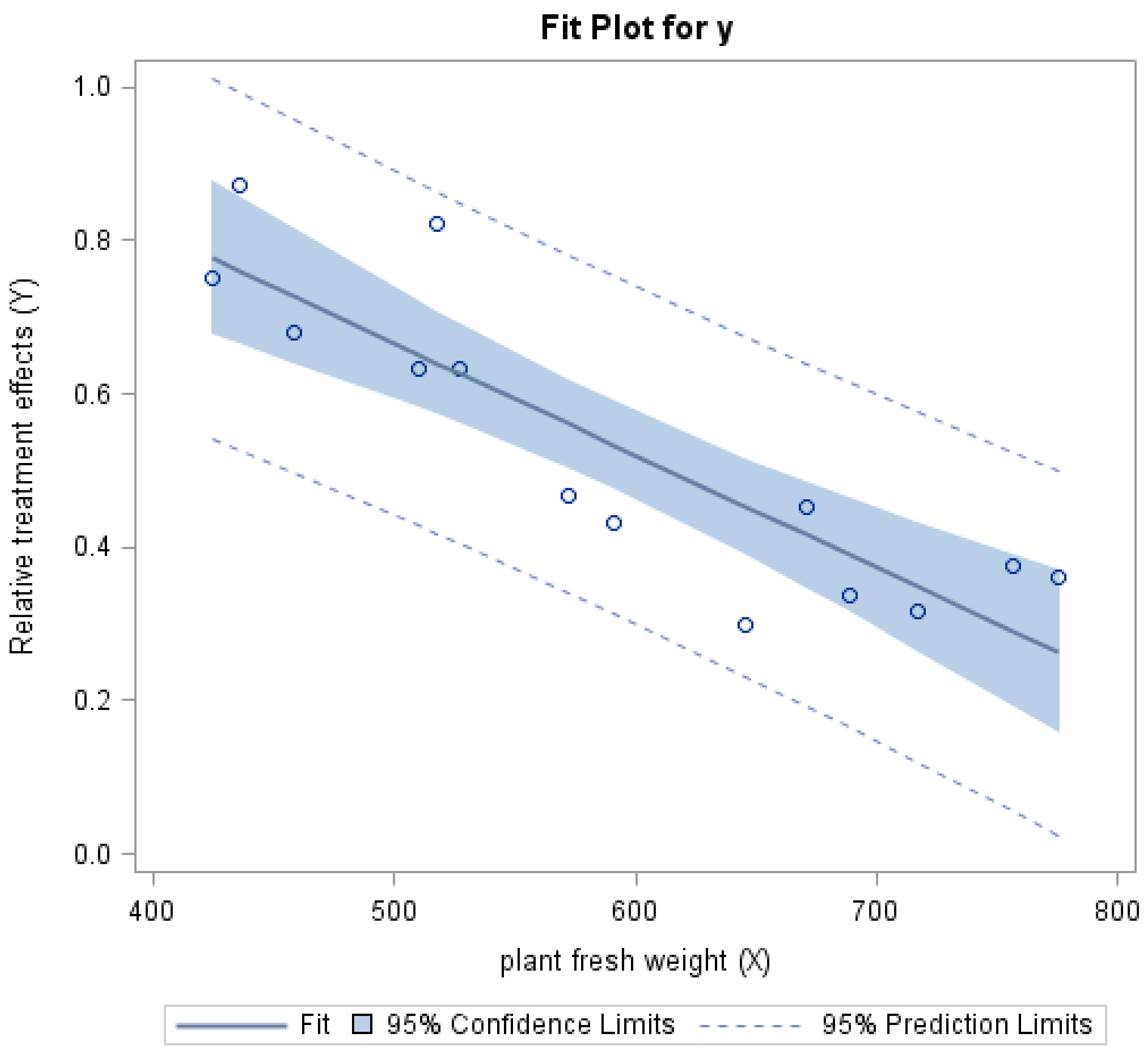


**Supplemental data II** Relationship between relative treatment effect of fungal colonization and fresh weight of *A. formosanus* Hayata after 120 days of co-culture. The estimated regression equation is $\hat{y}_{i}$ = 1.39822 - 0.00146 x*_i_* (where *i* = 14). The coefficient of determination (r^2^) is 0.7747, and the correlation coefficient (r) is -0.8801 and indicates a negative correlation between plant fresh weight (variable X) and treatment (Variable Y). This experiment was repeated twice.

**Testing for significance**

1. Using t test : Null hypothesis H_0_: β1 = 0; Alternative hypothesis H_1_: β1 ≠ 0
2. Confidence level (1-α) = 0.95, α = 0.05
3. Rejection region: a two-tailed test
4. Student’s t distribution, lTl > t _(1-α/2 = 0.025, df = n-2 =12)_ = 2.179 or *P*-value < 0.05

lTl = 6.43 > t _(0.025, 12)_ = 2.179, and *P*-value < 0.05; therefore H_0_ is rejected

1. H_1_ is accepted, which means each additional unit (x) will decrease 0.00146 in y.
2. Using F test : Null hypothesis H_0_: β1 = 0; Alternative hypothesis H_1_: β1 ≠ 0
3. Confidence level (1-α) = 0.95, α = 0.05
4. Rejection region: a one-tailed test

F > F _(1-α = 0.95, df1 = 1, df2 = 12)_ = 4.75 and P value < 0.05

1. F = 41.25 > 4.75, and *P*-value < 0.05; therefore H_0_ is rejected
2. H_1_ is accepted which means each additional unit (x) will decrease 0.00146 in y.
